# Plasma DNA Integrity as a Prognostic Biomarker for Colorectal Cancer Chemotherapy

**DOI:** 10.1155/2021/5569783

**Published:** 2021-05-26

**Authors:** Feng Zhu, Jing Ma, Dongdong Ru, Ningning Wu, Yunhua Zhang, Huiyuan Li, Xiaoli Liu, Jianfeng Li, Huiling Zhang, Yue Xu, Jiangman Zhao, Hui Tang, Yusheng Wang, Weihua Fu

**Affiliations:** ^1^Department of General Surgery, Tianjin Medical University General Hospital, Tianjin 300052, China; ^2^Department of General Surgery, Jincheng People's Hospital, Jincheng 048026, China; ^3^Shanghai Biotecan Pharmaceuticals Co., Ltd., 180 Zhangheng Road, Shanghai 201204, China; ^4^Shanghai Zhangjiang Institute of Medical Innovation, Shanghai 201204, China

## Abstract

**Objectives:**

To verify whether the concentrations and integrity index of circulating cell-free DNA (cfDNA) in serum may be clinically useful for the progression monitoring of colorectal cancer (CRC) patients.

**Methods:**

Serum samples were collected from 76 primary CRC patients who underwent surgery, including 60 with chemotherapy and 43 with follow-up. Long (247 bp) and short (115 bp) DNA fragments in serum were detected by real-time quantitative PCR by amplifying the ALU repeats. Ten serum traditional biomarkers levels were detected by chemiluminescence immunoassay assay.

**Results:**

The median DNA integrity index (ALU247/ALU115) of serum DNA in the preoperative group was significantly higher than those in the postchemotherapy and the follow-up groups, while cfDNA concentration (ALU115) was significantly lower in the preoperative group compared with the postchemotherapy and the follow-up groups. CEA and CA242 were significantly lower in the postoperative group than in the preoperative group.

**Conclusions:**

Serum DNA integrity index (ALU247/115) may prove to be a promising candidate biomarker for prognostic prediction of CRC who underwent chemotherapy and during short-term follow-up.

## 1. Introduction

Colorectal cancer (CRC), a common malignant tumor of the digestive tract, presents with significant morbidity and mortality worldwide [1], with an annual incidence of one million cases and an annual mortality of more than 500,000 cases [2, 3]. Especially in Chinese major cities, the incidence of CRC is increasing every year [4]. Furthermore, the overall survival of CRC tends to be poor, and approximately 50% of CRC patients ultimately died from distant metastasis [5]. Therefore, CRC treatment in early detection and diagnosis is vital important.

Tumor markers are widely applied to evaluate tumor diagnosis, treatment, and prognosis. Multiple serum markers including carcinoembryonic antigen (CEA), carbohydrate antigen 19-9 (CA19-9), carbohydrate antigen 242 (CA242) [6], and alpha-fetoprotein (AFP) [7] have been well recognized as tumor markers for CRC, as well as neuron specific enolase (NSE) [8], cytokeratin 19 (CK19) [9], squamous cell carcinoma antigen (SCC) [10], complexed prostate-specific antigen (c-PSA) [11], carbohydrate antigen 125 (CA125) [12], and carbohydrate antigen 72-4 (CA72-4) [13]. However, not all CRC cases can be diagnosed by CEA or CA19-9 alone owing to unstable detection and incremental concentrations in benign diseases [14], and CEA only has a sensitivity of 43% [15]. Due to poor sensitivity, its therapeutic effect of single application is not significant, so it is frequently used in combination with other tumor markers. Therefore, there is an urgent need to find a noninvasive biomarker that can be commonly applied for screening diagnosis, adjuvant detection of recurrence, or monitoring of metastatic CRC.

A promising approach is the quantification of tumor-related plasma cell-free DNA (cfDNA), which has shown potential in patients' cancer detection [16, 17] and prognosis [18]. CfDNA has been extensively studied in many cancers-associated cfDNA molecular characteristics, including copy number aberrations, methylation changes, single-nucleotide mutations, cancer-derived viral sequences, and chromosomal rearrangements [19].

Recently, cfDNA concentration and cfDNA integrity, which represent the quantity and quality of cfDNA, respectively, have been investigated as diagnostic or prognostic markers in many cancers in a large number studies [20–22]. Elevated cfDNA concentrations have been shown in many cancers compared to healthy controls [19, 23]. CfDNA integrity is calculated as the ratio of longer DNA fragment concentration to shorter ones of a specific genetic locus and indicates the extent of cfDNA fragmentation.

In the present study, we sought to identify the function of cfDNA concentration and cfDNA integrity as well as traditional biomarkers in CRC. Blood samples were collected from primary CRC patients at the following times: the pre and postoperative (76 CRC patients), the 3 cycles of chemotherapy (60 CRC patients), and the follow-up (43 CRC patients). Our aim was to compare total levels of cfDNA concentration, cfDNA integrity, and traditional biomarkers among the pre and postoperative, the postchemotherapy, and the follow-up groups of patients with CRC to see whether serum cfDNA or traditional biomarkers could predict the prognosis of CRC.

## 2. Materials and Methods

### 2.1. Subjects and Sample Collection

Total 76 primary CRC patients were recruited from Jincheng People's Hospital from April 2018 to October 2019. All 76 primary CRC patients received surgery, and 60 patients among them received both surgery and the 3 cycles of chemotherapy. At postoperative 6 months, 43 patients among them who received both surgery and the 3 cycles of chemotherapy were followed-up. No signs of recurrence or metastasis was assessed by computed tomography at 6 months after the operation in any of the patients.

Plasma samples were collected at 6 time points, including the preoperative (the first time, 7 days before surgery), the postoperative (the second time, 3 weeks after surgery), the first cycle of chemotherapy (the third time, approximately 6 weeks after surgery), the second cycle of chemotherapy (the fourth time, approximately 3 weeks after cycle 1), the third cycle of chemotherapy (the fifth time, approximately 3 weeks after cycle 2), and the follow-up (the sixth time, at the postoperative for 6 months). All procedures were performed in accordance with the ethical standards of the Clinical Research Ethics Committee of the Jincheng People's Hospital, and written informed consent was obtained from all participants included in the study.

### 2.2. Blood Collection and cfDNA Isolation

Ten ml venous blood was collected in an ethylenediamine tetraacetic acid (EDTA) tube and stranded still 20 min in room temperature. To ensure cell-free plasma collection, all blood samples were centrifuged in 3000 rpm for 10 min and then 13,000 rpm for 10 min. CfDNA was extracted from plasma samples using the QIAamp Circulating Nucleic Acid Kit (Qiagen, Hilden, Germany) according to the manufacturer's protocol. DNA was then stored at −80°C until further analysis. Quantification of cfDNA was performed using the quantitative PCR (qPCR) method [24].

### 2.3. Real-Time Alu-PCR

The primer ALU115 amplified the shorter (115 bp) fragments and the primer ALU247 amplified the longer (247 bp) fragments. Amplifying and quantifying shorter and longer fragments from abundant genomic ALU repeats according to a previous study by qPCR [16]. The ALU-qPCR result obtained with ALU115 primer represents the total quantity of cfDNA. DNA integrity index was calculated as the ratio of ALU-qPCR result (ALU247/115). The sequences of the primers were as follows: ALU115: forward, 5′-CCTGAGGTCAGGAGTTCGAG-3′ and reverse, 5′-CCCGAGTAGCTGGGATTACA-3′; ALU247: forward, 5′-GTGGCTCACGCCTGTAATC-3′ and reverse, 5′-CAGGCTGGAGTGCAGTGG-3′.

The reaction mixture for each ALU-qPCR contained 5 ml DNA template, 0.5 ml of each forward and reverse primer (ALU115 or ALU247), 10 ml SYBR Green Master Mix (Rox, Weitefeld, Germany), and 4 ml double-distilled water in a total reaction volume of 20 ml with 95°C for 10 min, followed by 35 cycles of 95°C for 15 s, and annealing at 64°C for 1 min in the 7500 Real-Time PCR System (ABI, Abilene, TX, USA). Each PCR assay included a plasma DNA sample, a water template as negative control, and a human genomic DNA as positive control. All reactions were carried out in duplicates. Researchers performing the qPCR assays were blinded to the patient's clinical outcomes.

### 2.4. Quantification of Traditional Serum Biomarkers

The concentrations of serological tumor markers, including CA19-9, CA72-4, CA125, CA242, CEA, AFP, c-PSA, CK19, NSE, and SCC, were determined in the Clinical Pathology Laboratory of the Jincheng People's Hospital according to the standard protocols.

### 2.5. Statistical Analysis

Statistical analysis was performed using GraphPad Prism 6.0 (GraphPad Software, La Jolla, CA, USA) or SPSS19.0 program (SPSS Inc., Chicago, IL, USA). Parametric statistics (*t*-test) were used for normally distributed data, and nonparametric statistics including the Mann–Whitney test for unpaired two groups or the Wilcoxon test for paired two groups were used for nonnormally distributed data. Spearman's correlation analysis was applied to analyze the correlation between different biomarkers. A *p* value <0.05 was considered statistically significant.

## 3. Results

### 3.1. Characteristics of Primary CRC Patients

Serum samples were enrolled from a total of surgical 76 primary CRC patients. An analysis on ALU115 and ALU247/115 in different subgroups of CRC patients was with respect to gender, age, tumor size, smoking, histologic differentiation, and tumor-node-metastasis (TNM) stage. The results indicated that only ALU115 was correlated with age (*p*=0.024) (Table S1).

### 3.2. Comparison of Serum ALU115, ALU247/115, and Clinical Biomarkers in Primary CRC Patients between Preoperative and Postoperative Groups

**Figure 1:**
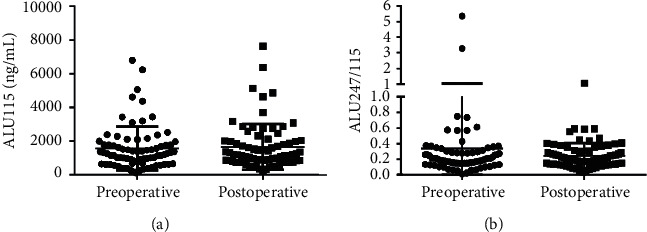
Scatter plots of ALU115 and ALU247/115 in serum from primary CRC patients between the preoperative group and the postoperative group. (a), (b) ALU115 and ALU247/115 were determined by ALU-qPCR.

Serum ALU115 and ALU247/115 levels were determined in the 76 primary CRC patients between the pre and postoperative groups. The median ALU115 was 1250 (662.5–1773) ng/*μ*l and ALU247/115 was 0.198 (0.149–0.295) in the preoperative group; at the postoperative for 21 days, the median ALU115 and ALU247/115 was 1190 (791.3–1984) ng/*μ*l and 0.200 (0.137–0.306), respectively, in the postoperative group. However, there were no statistical differences in ALU115 and ALU247/115 of serum DNA between the two groups (Figure 1). On the contrary, Hao et al. reported that both ALU115 and ALU247/115 were increased before surgery and decreased significantly after surgery in 20 surgical CRC patients [24]. Therefore, large studies need to verify this issue in near future.

**Figure 2:**
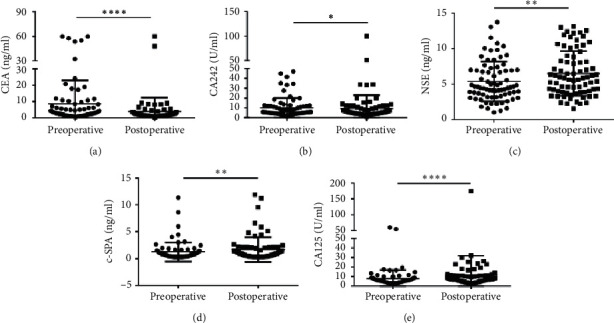
Scatter plots of the cancer biomarkers level in serum from primary CRC patients between the preoperative group and the postoperative group. (a–e) The Wilcoxon signed rank test was used to assess the CEA (a), CA242 (b), NSE (c), c-SPA (d), and CA125 (e) levels between the two groups. ^*∗*^*p* < 0.05, ^*∗∗*^*p* < 0.01, and ^*∗∗∗∗*^*p* < 0.0001.

In addition, we selected 10 clinical biomarkers (CEA, NSE, CA19-9, CA242, CA72-4, AFP, SCC, c-SPA, CA125, and CK19) which were reported to be associated with CRC progress. The median serum CEA concentration was 3.110 (1.410–8.000) ng/ml in the preoperative group vs. 1.835 (1.205–2.885) ng/ml in the postoperative group, showing a significant difference between the two groups (*p* < 0.00001) (Figure 2(a)). Similarly, Hu et al. reported that CEA was decreased notably in CRC patients after operations [25]. The median of CA242 concentration was also significant decreased in the postoperative group (5.500 (3.908–9.753) U/ml) than in the preoperative group (5.865 (1.410–8.000) U/ml) (*p* < 0.00001) (Figure 2(b)). However, Peng et al. reported that CA242 showed an insignificant difference between the metastasis/local recurrence group and the nonmetastasis/local recurrence group after curative resection [26]. In our present study, we found that CA242 was significantly decreased in primary CRC patients after surgery. However, the median concentrations of NSE (4.585 (3.355-6.980) ng/ml) vs. (5.855 (3.880-8.690) ng/ml), c-SPA (0.715 (0.393-1.318) ng/ml) vs. (0.895 (0.413-1.960) ng/ml), and CA125 (5.730 (4.125-8.200) U/ml) vs. (7.040 (5.253-11.160) U/ml) were significant lower in the preoperative group than in the postoperative group (Figures 2(c)–2(e)). Other 5 clinical biomarkers showed no statistical differences between the two groups (Figure S1). These results indicated that CEA and CA242 could be used as a prognostic indicator in CRC patients who underwent surgery.

### 3.3. Correlation between ALU115, ALU247/115, and Clinical Biomarkers at Baseline

Serum ALU115 and ALU247/115 levels and 10 clinical biomarkers were determined in 76 primary CRC patients before surgery. To evaluate the correlations among ALU115, ALU247/115, and 10 traditional biomarkers, Spearman's correlation analysis was applied. However, neither ALU115 (Figure S2) nor ALU247 (Figure S3) has significant correlations with the 10 clinical biomarkers. It was suggested that ALU115 and ALU247/115 were relatively independent prognostic indicators in CRC.

### 3.4. Comparison of Serum ALU115, ALU247/115, and Clinical Biomarkers in Primary CRC Patients from Preoperative to Postchemotherapy

**Figure 3:**
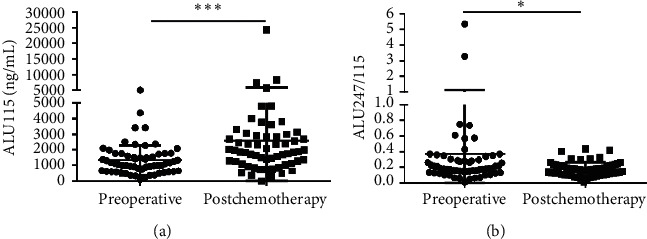
Scatter plots of ALU115 and ALU247/115 in serum from primary CRC patients between the preoperative group and the postchemotherapy group. (a), (b) ALU115 and ALU247/115 were determined by ALU-qPCR. ^*∗*^*p* < 0.05 and ^*∗∗∗*^*p* < 0.001.

CfDNA was measured in 60 of 76 primary CRC who received both surgery and the 3 cycles of chemotherapy. The median ALU115 and ALU247/115 was 1128 (637.5–1741) ng/*μ*l and 0.185 (0.139–0.303), respectively, in the preoperative group; at postchemotherapy (which refers to the patients who had undergone the 3 cycles of chemotherapy), the median ALU115 was 1865 (1168–2884) ng/*μ*l and ALU247/115 was 0.168 (0.115–0.223). Significant differences were observed in ALU115 and ALU247/115 of serum DNA between the two groups (Figure 3). The results indicated that the level of ALU247/115 was significantly lower, while ALU115 was noticeably higher in the postchemotherapy group than in the preoperative group.

**Figure 4:**
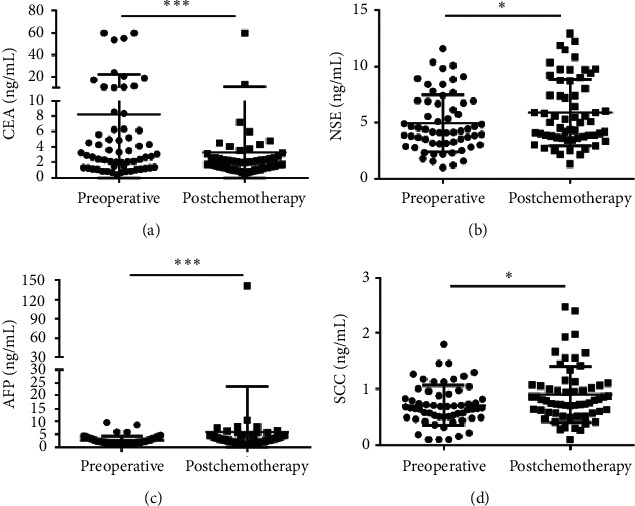
Scatter plots of the cancer biomarkers level in serum from primary CRC patients between the preoperative group and the postchemotherapy group. (a–d) The Wilcoxon signed rank test was used to assess the CEA (a), NSE (b), AFP (c) and SCC (d) levels between the two groups. ^*∗*^*p* < 0.05 and ^*∗∗∗*^*p* < 0.001.

In addition, the median serum CEA concentration was 2.980 (1.340–6.293) in the preoperative group vs. 1.750 (1.310–2.578) ng/ml in the postchemotherapy group, showing a significant difference in the two groups (*p*=0.0006) (Figure 4(a)). However, no significant change could be detected in CEA levels during adjuvant chemotherapy in stage III colon cancer [27]. Therefore, large-scale studies need to verify these results in the future. In addition, the median concentrations of NSE (4.215 (3.140–6.843) ng/ml) vs. (5.040 (3.705–8.533) ng/ml), AFP ((2.220 (1.528–3.240) ng/ml) vs. (3.030 (1.985–4.950) ng/ml)), and SCC (0.660 (0.493–0.903) ng/ml) vs. (0.795 (0.573–1.068) ng/ml) were significantly lower in the preoperative group than in the postchemotherapy group (Figures 4(b)–4(d)). Other 6 clinical biomarkers showed no statistical differences between the two groups (Figure S4).

**Figure 5:**
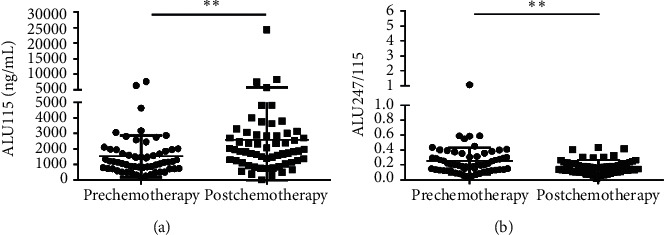
Scatter plots of ALU115 and ALU247/115 in serum from primary CRC patients between the prechemotherapy group and the postchemotherapy group. (a), (b) ALU115 and ALU247/115 were determined by ALU-qPCR. ^*∗∗*^*p* < 0.01.

To evaluate the only treatment of chemotherapy in 60 CRC patients, we compared the level of ALU115 and ALU247/115 between the prechemotherapy group (which is defined as the patients group who had taken the surgery but had not taken the chemotherapy yet) and the postchemotherapy group, and the median ALU115 and ALU247/115 was 1173 (736.3–1935) ng/*μ*l and 0.200 (0.1309–0.345), respectively, in the prechemotherapy group. After the 3 cycles of chemotherapy, the median ALU115 was 1865 (1168–2884) ng/*μ*l and ALU247/115 was 0.168 (0.115–0.223). Significant differences were observed in ALU115 and ALU247/115 of serum of DNA between the two groups (Figure 5). The results indicated that the level of ALU247/115 was significantly lower, while ALU115 was significantly higher in the postchemotherapy group. Similarly, Lehner et al. reported that ALU115 was slightly higher in the postchemotherapy group than in the prechemotherapy group in breast cancer, but with no significant difference [28]. Although chemotherapy should have induced cell death and release of DNA in the circulation, it is equally possible that the arrest of tumor cell proliferation also reduced DNA release. This increase may be because of the residual tumor tissue during the surgical procedure or postoperative inflammation or postchemotherapy with subsequent release of cfDNA. Another explanation may be that the release of the DNAse inhibitor from the tumor cells was reduced [29]. Although the mechanism of increasing cfDNA concentration is not clear at present, our findings suggest that ALU247/115 could be used as an indicator for identification of the prognosis of CRC patients who underwent chemotherapy.

**Figure 6:**
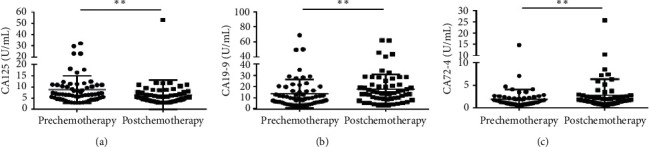
Scatter plots of ALU115 and ALU247/115 in serum from primary CRC patients between the prechemotherapy group and the postchemotherapy group. (a–c) The Wilcoxon signed rank test was used to assess the CA125 (a), CA19-9 (b), and CA72-4 (c) levels between the two groups. ^*∗∗*^*p* < 0.01.

The median serum CA125 concentration was 6.740 (4.860–10.870) ng/ml) in the prechemotherapy group and was significantly higher than in the postchemotherapy group (5.135 (3.615–7.585) ng/ml) (*p*=0.0018) (Figure 6(a)). This study is in accordance with the previous study that CA125 was significantly decreased after chemotherapy in CRC patients [30]. However, the median concentrations of CA19-9 are (9.750 (5.945–16.540) ng/ml) vs. (14.610 (9.398–21.840) ng/ml) and CA72-4 are (1.450 (0.920–2.170) ng/ml) vs. (1.670 (1.153–2.618) ng/ml) in the pre and postchemotherapy groups, respectively, all showing significantly higher concentrations in the postchemotherapy group (Figures 6(b) and 6(c)). Other biomarkers showed no statistical differences between the two groups (Figure S5). In this study, CEA no longer responds to chemotherapy treatment. It is suggested that CEA respond to surgery rather than chemotherapy, while CA125 may positively respond to chemotherapy.

### 3.5. Comparison of Serum ALU115, ALU247/115, and Clinical Biomarkers in Primary CRC Patients from Preoperative to Follow-Up

**Figure 7:**
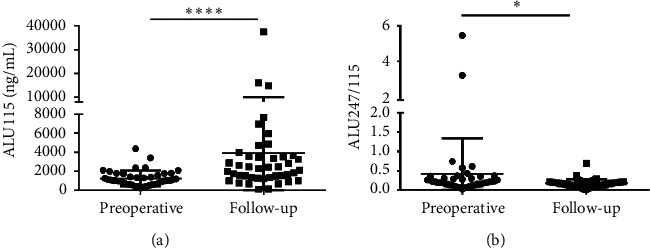
Scatter plots of ALU115 and ALU247/115 in serum from primary CRC patients between the preoperative group and the follow-up group. (a), (b) ALU115 and ALU247/115 were determined by ALU-qPCR. ^*∗*^*p* < 0.05, and ^*∗∗∗*^*p* < 0.001.

At the postoperative for 6 months, total 43 CRC patients who received both surgery and the 3 cycles of chemotherapy were followed-up. The median ALU115 and ALU247/115 was 1015 (630–1715) ng/*μ*l and 0.212 (0.136–0.305), respectively, in the preoperative group; after the operative for 6 months, the median ALU115 and ALU247/115 was 2240 (1315–3565) ng/*μ*l and 0.163 (0.098–0.210), respectively, in the follow-up. Significant differences were observed in the ALU115 and ALU247/115 of serum DNA between the two groups (Figure 7). The results indicated that ALU247/115 was significantly lower, while ALU115 was significantly higher in the follow-up group than in the preoperative group.

**Figure 8:**
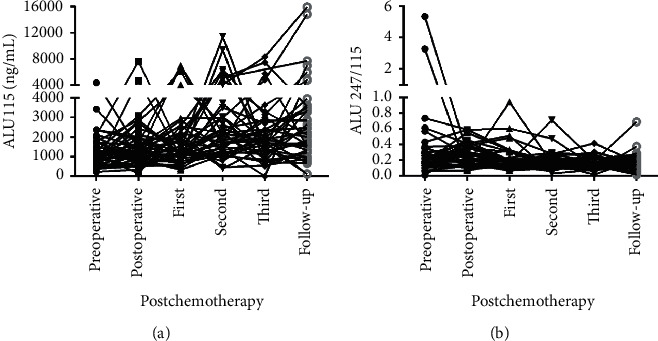
Line charts of the preoperative to the follow-up groups serum ALU115 and ALU247/115 in 43 CRC patients. (a), (b) ALU115 and ALU247/115 were determined by ALU-qPCR.

A dynamic diagram in Figure 8 illustrates ALU115 and ALU247/115 in periods of the pre and postoperative, the postchemotherapy, and the follow-up. There was a general trend that ALU115 was gradually higher from the preoperative to the follow-up periods, while ALU247/115 was significantly down from the postoperative to the follow-up periods. The follow-up data obtained from 43 CRC patients showed that there was a general trend of decrease in ALU247/115 rather than ALU115 after surgery and chemotherapy as compared with those before surgery, although the rate of decrease after surgery and chemotherapy was different in all cases. For instance, a linear or sharp decrease was observed in some cases, whereas there was an increase in some other cases at the first cycle chemotherapy, the second cycle of chemotherapy, or the follow-up. This increase may be because of the residual tumor tissue during the postoperative or postchemotherapy inflammation, with subsequent release of cfDNA.

**Figure 9:**
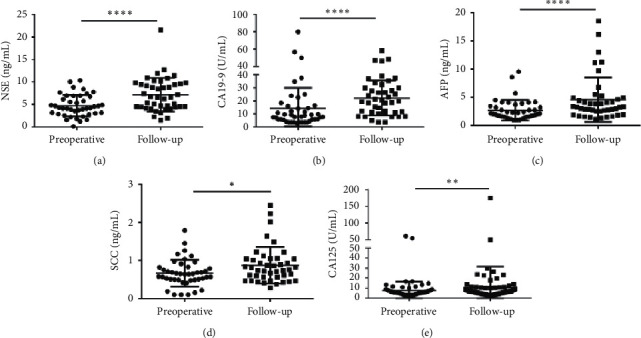
Scatter plots of the cancer biomarkers level in serum from primary CRC patients between the preoperative group and the follow-up group. (a–e) The Wilcoxon signed rank test was used to assess the NSE (a), CA19-9 (b), AFP (c), SCC (d), and CA125 (e) levels between the two groups. ^*∗*^*p* < 0.05, ^*∗∗*^*p* < 0.05, and ^*∗∗∗∗*^*p* < 0.0001.

Besides that, the median concentrations of NSE (4.215 (2.985–6.768) vs. 6.590 (4.430–9.570)), CA19-9 (7.940 (5.310–15.870) vs. 19.550 (11.020–30.030)), AFP (2.140 (1.550–3.460) vs. 3.310 (2.540–4.940)), SCC (0.640 (0.490–0.820) vs. 0.760 (0.560–1.040)) and CA125 (5.845 (4.125–7.538) vs. 6.550 (4.438–9.930)) were significantly lower in the preoperative group than in the follow-up (Figure 9). CEA and CA242 showed no responses to the follow-up (Figure S6), while the level CA125 was higher in the follow-up group than in the preoperative group. The results indicated that CEA, CA242, and CA125 were not suitable to predicate the CRC during follow-up.

## 4. Discussion

CfDNA, as an emerging biomarker, has practical advantages, including high sensitivity, noninvasiveness, and repeatability. It has been hypothesized that cfDNA may be a predictive factor of tumor response and a good candidate for a prognostic factor. The ALU sequences, typically 300 nucleotides in length, are the most abundant and active repeated elements in the human genome, accounting for more than 10% of the genome [31, 32]. ALU sequences can be used for sensitive quantification of human genomic DNA in neoplastic specimen extracts [33, 34].

In the present study, we measured the serum ALU115 and ALU247/115 dynamics in surgical 76 primary CRC patients. To be specific, first of all, only ALU115 was found to be significant correlated with age in CRC patients. Similarity, Jylhava et al. reported that plasma cfDNA concentration level was significant higher in the older women than the younger controls [35]. On the contrary, Sozzi et al. demonstrated a significant positive relationship between age and cfDNA concentration in an equal number of nonsmall cell lung cancer patients and healthy controls [36]. Therefore, a large case-control matched study needs addressing this issue. Second, both the levels of ALU115 and ALU247/115 showed no significant differences between the pre and postoperative groups. However, Hao et al. reported that both ALU115 and ALU247/115 were significantly decreased after surgery in CRC patients [24]. A large number of studies need to demonstrate this issue in the future. Third, after being treatment with the 3 cycles of chemotherapy, the levels of ALU247/115 were significantly lower in those postchemotherapy and follow-up CRC patients, while the levels of ALU115 were noticeably higher in both the postchemotherapy and follow-up groups. Last but not least, biomarkers CEA and CA242 showed a good performance after surgery, and CA125 may be a potential biomarker for only chemotherapy treatment.

Several reports have indicated higher levels of total cfDNA concentration in the plasma of lung cancer patients compared with healthy controls, serving as a potential diagnostic biomarker [37, 38]. However, Tissot et al. reported that total cfDNA concentration is not associated with chemotherapy response [39]. A large number of studies reported higher DNA integrities in plasma of patients with ovarian, breast, and colorectal cancer as compared with healthy controls [16, 40]. In breast cancer, increased integrity of serum DNA has been correlated to a worse disease outcome and poor response to adjuvant chemotherapy [16, 41]. However, other reports could not find a difference of DNA integrity values in the same tumor types [42–44]. In this study, detection of changes in the serum concentration of ALU247/115 may prove useful for dynamic monitoring of the prognosis of CRC patients who underwent chemotherapy and during follow-up.

There were some limitations in this study. First, the sample size used was relatively small, and no healthy controls were recruited in this study, so we cannot compare primary CRC patients with health controls on the baseline. Second, no recurrence or metastasis CRC patients were found during short-term follow-up, so we cannot compare the ALU115, ALU247/115, or traditional biomarkers with each other to predict CRC patients' recurrence or metastasis. Thus, more work is required before ALU247/115 can be applied in the prognosis for CRC after chemotherapy and follow-up, and these findings should be validated in a larger cohort.

## 5. Conclusions

In this study, we observed decreased cfDNA integrity in therapy CRC compared to primary CRC, and cfDNA integrity was able to perform as a prognostic marker independent of cfDNA. Our data demonstrate the potency of cfDNA integrity as a diagnostic biomarker by a comprehensive analysis of CRC chemotherapy patients and during follow-up. Furthermore, larger-scale and longer prospective studies are needed to confirm the clinical utility of cfDNA integrity in the prognostic prediction of CRC.

## Data Availability

The data used to support the findings of this study are included within the article.

## Supplementary Materials

Supplementary Materials Figure S1. Scatter plots of the cancer biomarkers level in serum from primary CRC patients between the preoperative group and the postoperative group. (a–e) The Wilcoxon signed rank test was used to assess the CA19-9 (a), CA72-4 (b), AFP (c), SCC (d), and CK19 (e) levels between the two groups. Figure S2. Spearman correlation analysis of the clinical cancer biomarkers level and ALU115. Spearman correlation analysis of (a) CEA and ALU115, (b) NSE and ALU115, (c) CA19-9 and ALU115, (d) CA242 and ALU115, (e) AFP and ALU115, (f) SCC and ALU115, (g) c-SPA and ALU115, (h) CK19 and ALU115, (i) CA72-4 and ALU115, and (j) CA125 and ALU115. Figure S3. Spearman correlation analysis of the clinical cancer biomarkers level and ALU247/115. Spearman correlation analysis of (a) CEA and ALU247/115, (b) NSE and ALU247/115, (c) CA19-9 and ALU247/115, (d) CA242 and ALU247/115, (e) AFP and ALU247/115, (f) SCC and ALU247/115, (g) c-SPA and ALU247/115, (h) CK19 and ALU247/115, (i) CA72-4 and ALU247/115, and (j) CA125 and ALU247/115. Figure S4. Scatter plots of the cancer biomarkers level in serum from primary CRC patients between the preoperative group and the postchemotherapy group. (a–d) The Wilcoxon signed rank test was used was used to assess the CA19-9 (a), CA242 (b), CA72-4 (c), c-SPA (d), CA125 (e), and CK19 (f) levels between the two groups. Figure S5. Scatter plots of the cancer biomarkers level in serum from primary CRC patients between the prechemotherapy group and the postchemotherapy group. (a–g) The Wilcoxon signed rank test was used to assess the CEA (a), NSE (b), CA242 (c), AFP (d), SCC (e), c-SPA (f), and CK19 (g) levels between the two groups. Figure S6. Scatter plots of the cancer biomarkers level in serum from primary CRC patients between the preoperative group and the follow-up group. (a–e) The Wilcoxon signed rank test was used to assess the CEA (a), CA242 (b), CA72-4 (c), c-SPA (d), and CK19 (e) levels between the two groups. Table S1. ALU115 and ALU247/115 of serum DNA in subgroups of primary CRC.
