# Supplementary material for: Plasma DNA Integrity as a Prognostic Biomarker for Colorectal Cancer Chemotherapy
Source: J Oncol. 2021 May 26;2021:5569783. doi: 10.1155/2021/5569783 (PMC8175143; doi:10.1155/2021/5569783)
Supplement: Supplementary Materials — Figure S1. Scatter plots of the cancer biomarkers level in serum from primary CRC patients between the preoperative group and the postoperative group. (a–e) The Wilcoxon signed rank test was used to assess the CA19-9 (a), CA72-4 (b), AFP (c), SCC (d), and CK19 (e) levels between the two groups. Figure S2. Spearman correlation analysis of the clinical cancer biomarkers level and ALU115. Spearman correlation analysis of (a) CEA and ALU115, (b) NSE and ALU115, (c) CA19-9 and ALU115, (d) CA242 and ALU115, (e) AFP and ALU115, (f) SCC and ALU115, (g) c-SPA and ALU115, (h) CK19 and ALU115, (i) CA72-4 and ALU115, and (j) CA125 and ALU115. Figure S3. Spearman correlation analysis of the clinical cancer biomarkers level and ALU247/115. Spearman correlation analysis of (a) CEA and ALU247/115, (b) NSE and ALU247/115, (c) CA19-9 and ALU247/115, (d) CA242 and ALU247/115, (e) AFP and ALU247/115, (f) SCC and ALU247/115, (g) c-SPA and ALU247/115, (h) CK19 and ALU247/115, (i) CA72-4 and ALU247/115, and (j) CA125 and ALU247/115. Figure S4. Scatter plots of the cancer biomarkers level in serum from primary CRC patients between the preoperative group and the postchemotherapy group. (a–d) The Wilcoxon signed rank test was used was used to assess the CA19-9 (a), CA242 (b), CA72-4 (c), c-SPA (d), CA125 (e), and CK19 (f) levels between the two groups. Figure S5. Scatter plots of the cancer biomarkers level in serum from primary CRC patients between the prechemotherapy group and the postchemotherapy group. (a–g) The Wilcoxon signed rank test was used to assess the CEA (a), NSE (b), CA242 (c), AFP (d), SCC (e), c-SPA (f), and CK19 (g) levels between the two groups. Figure S6. Scatter plots of the cancer biomarkers level in serum from primary CRC patients between the preoperative group and the follow-up group. (a–e) The Wilcoxon signed rank test was used to assess the CEA (a), CA242 (b), CA72-4 (c), c-SPA (d), and CK19 (e) levels between the two groups. Table S1. ALU115 and ALU247/115 of serum [file 5569783.f1.pdf]

Table S1. **ALU115 and ALU247/115 of serum DNA in subgroups of primary CRC patients.**

Clinical characteristics	No	cfDNA (ng/ml) (IQR 25–75)	*p*	ALU115（ng/ml) (IQR 25–75)	*p*	ALU247/ALU115 (IQR 25–75)	*p*
ALL	76						
Sex			0.056		0.253		0.831
Male	49	784 (569-1250)		1015（625-1865）		0.206 (0.142-0.284)	
Female	27	1170 (712-1660)		1455（1010-1765）		0186 (0.150-0.348)	
Age (years)			0.390		0.024*		0.977
≤60	29	862 (620-1395)		975 (560-1583)		0.195 (0.152-0.301)	
>60	47	966 (600-1420)		1295 (955-2150)		0.200 (0.133-0.297)	
smoking			0.115		0.052		0.499
Yes	20	771 (483-1298)		947.5 (536.3-1499)		0.211 (0.155-0.329)	
No	56	976 (683-1508)		1378 (906.3-1974)		0.185 (0.147-0.289)	
Tumour size (cm)			0.246		0.240		0.107
≤5	49	848 (581-1430)		1145 (665-1663)		0.184 (0.131-0.280)	
>5	27	1040 (732-1410)		1480 (630-2370)		0.217 (0.160-0.360)	
TNM			0.323		0.468		0.337
I-II	47	848 (582-1330)		1180 (630-1750)		0.215 (0.150-0.297)	
III-IV	38	1026 (619.5-1773)		1430 (667.5-2355)		0.173 (0.134-0.308)	
Histologic differentiation			0.245		0.250		0.652
Low	10	914 (612-1463)		1300 (872.5-2011)		0.190 (0.126-0.266)	
Middle	57	1030 (613-1500)		1325 (665-2023)		0.206 (0.150-0.311)	
High	9	766 (464-898)		940 (592.5-1323)		0.200 (0.127-0.354)	

*P<0.05 was considered significant; IQR, interquartile range; TNM, tumour node metastasis.

FIGURE S1

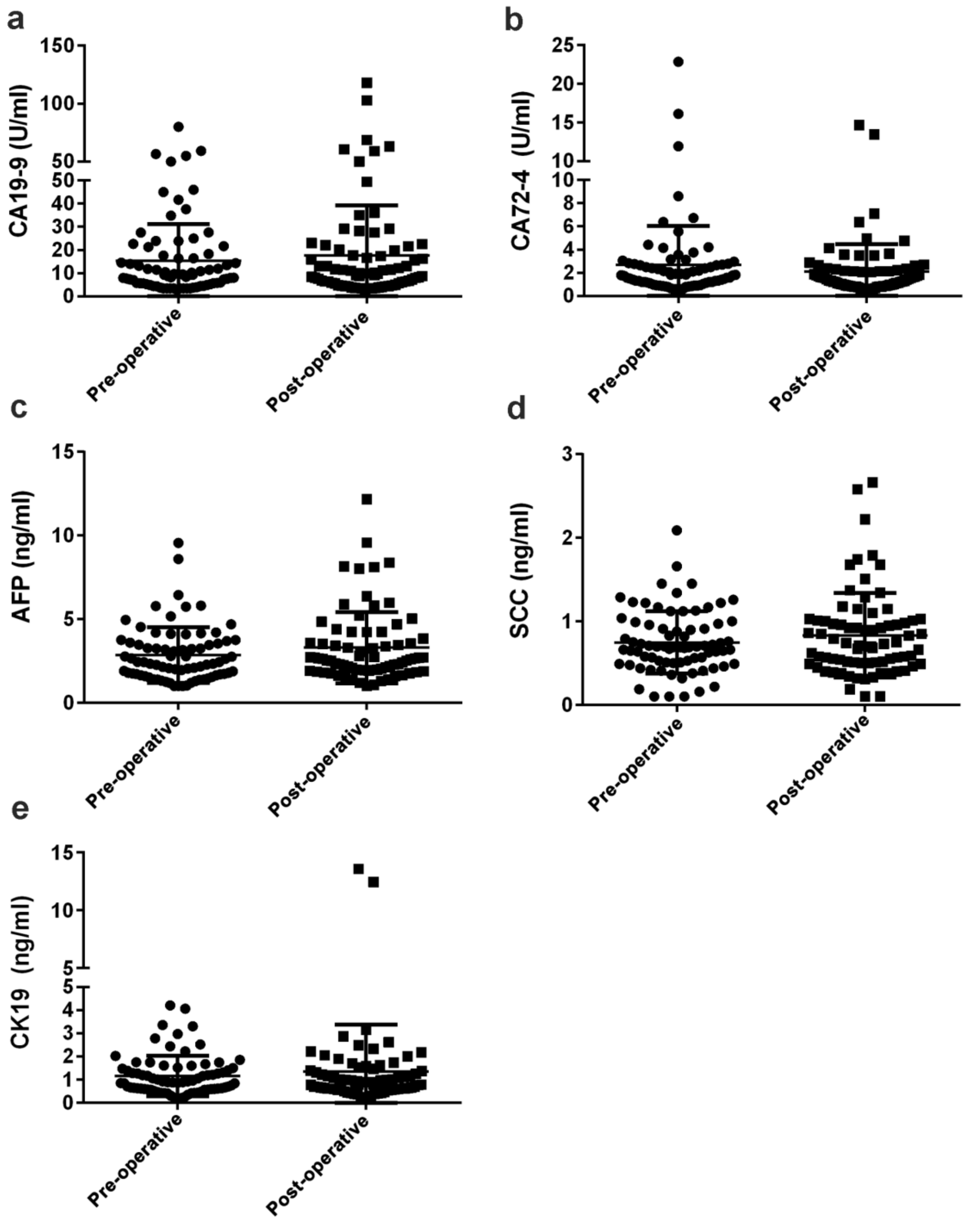

a
CA19-9 (U/ml)
150
100
50
50
40
30
20
10
0
Pre-operative
Post-operative
b
CA72-4 (U/ml)
25
20
15
10
10
8
6
4
2
0
Pre-operative
Post-operative
c
AFP (ng/ml)
15
10
5
0
Pre-operative
Post-operative
d
SCC (ng/ml)
3
2
1
0
Pre-operative
Post-operative
e
CK19 (ng/ml)
15
10
5
5
4
3
2
1
0
Pre-operative
Post-operative

FIGURE S2

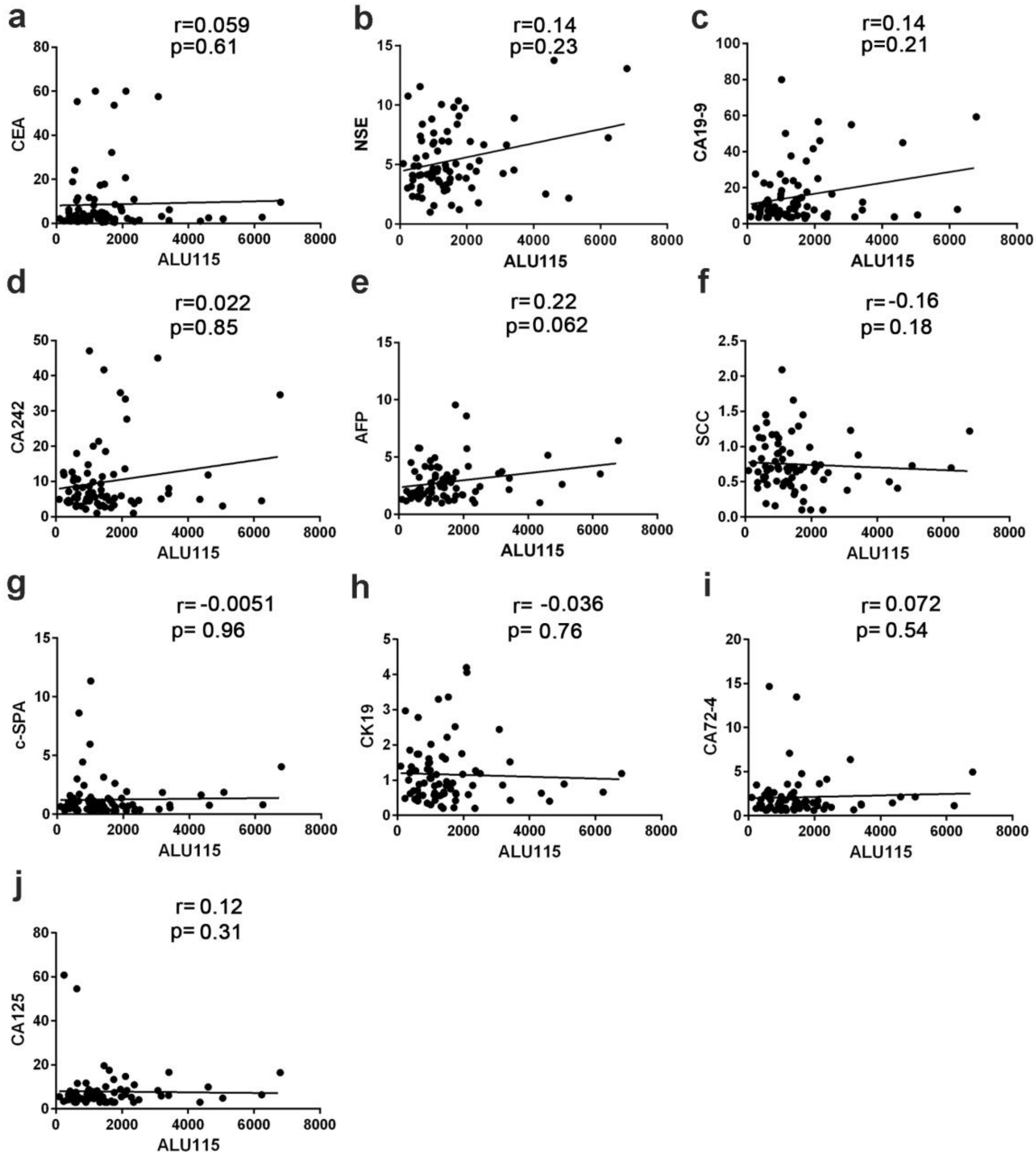

a
r=0.059
p=0.61
CEA
ALU115
b
r=0.14
p=0.23
NSE
ALU115
c
r=0.14
p=0.21
CA19-9
ALU115
d
r=0.022
p=0.85
CA242
ALU115
e
r=0.22
p=0.062
AFP
ALU115
f
r=-0.16
p=0.18
SCC
ALU115
g
r= -0.0051
p= 0.96
c-SPA
ALU115
h
r= -0.036
p= 0.76
CK19
ALU115
i
r= 0.072
p= 0.54
CA72-4
ALU115
j
r= 0.12
p= 0.31
CA125
ALU115

FIGURE S3

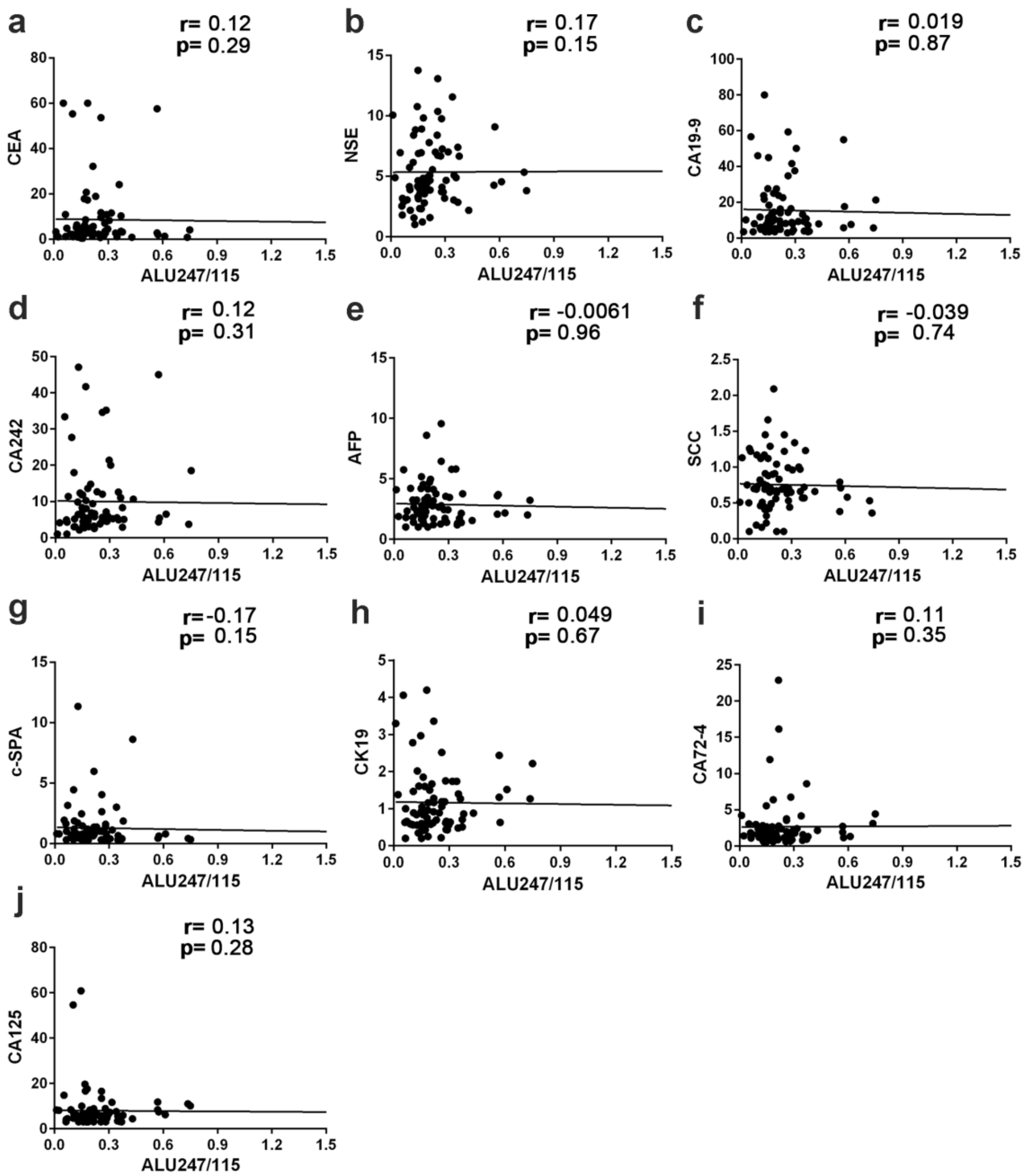
a
r= 0.12
p= 0.29
CEA
ALU247/115
b
r= 0.17
p= 0.15
NSE
ALU247/115
c
r= 0.019
p= 0.87
CA19-9
ALU247/115
d
r= 0.12
p= 0.31
CA242
ALU247/115
e
r= -0.0061
p= 0.96
AFP
ALU247/115
f
r= -0.039
p= 0.74
SCC
ALU247/115
g
r=-0.17
p= 0.15
c-SPA
ALU247/115
h
r= 0.049
p= 0.67
CK19
ALU247/115
i
r= 0.11
p= 0.35
CA72-4
ALU247/115
j
r= 0.13
p= 0.28
CA125
ALU247/115

FIGURE S4

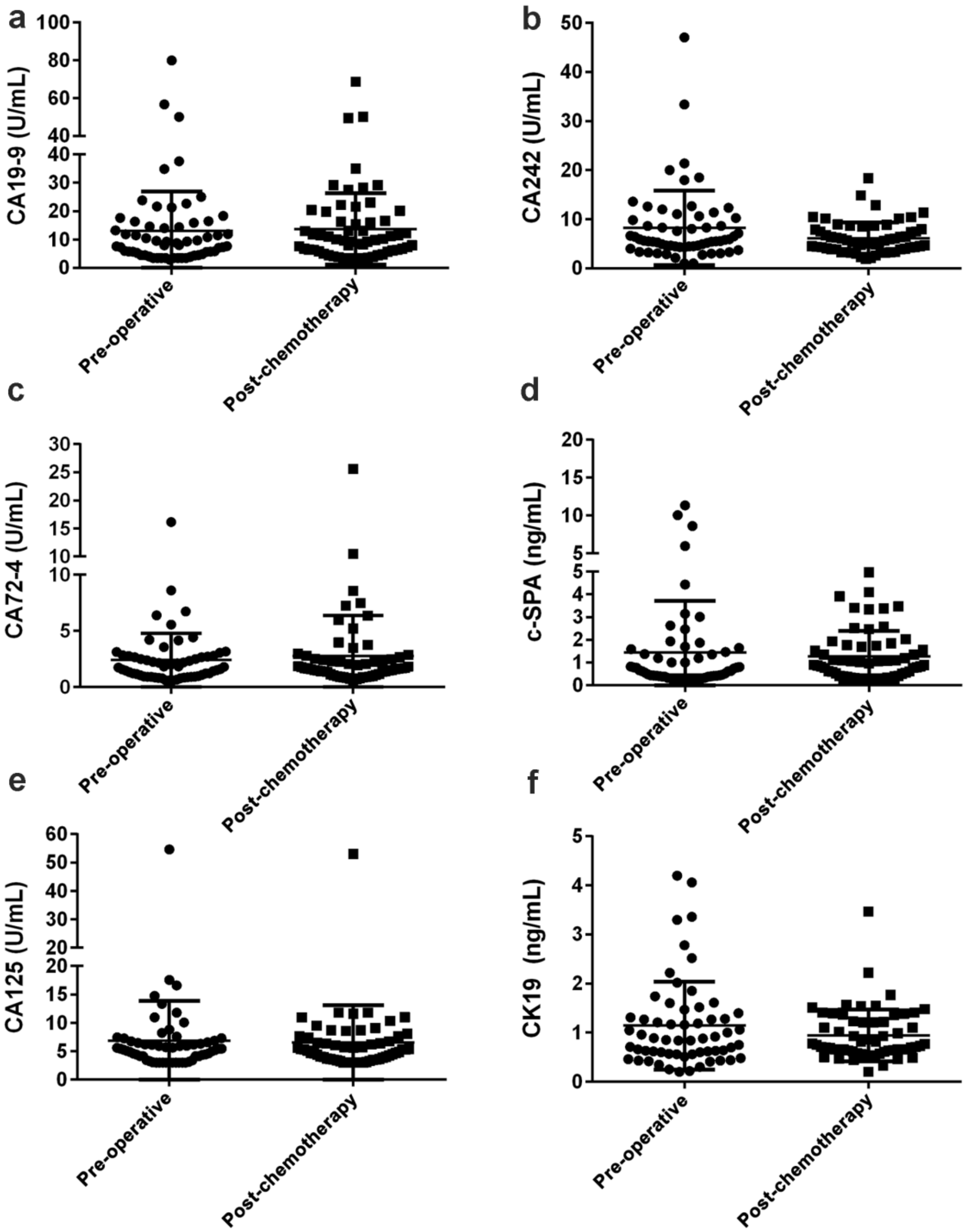

a
CA19-9 (U/mL)
100
80
60
40
40
30
20
10
0
Pre-operative
Post-chemotherapy
b
CA242 (U/mL)
50
40
30
20
10
0
Pre-operative
Post-chemotherapy
c
CA72-4 (U/mL)
30
25
20
15
10
10
5
0
Pre-operative
Post-chemotherapy
d
c-SPA (ng/mL)
20
15
10
5
5
4
3
2
1
0
Pre-operative
Post-chemotherapy
e
CA125 (U/mL)
60
50
40
30
20
20
15
10
5
0
Pre-operative
Post-chemotherapy
f
CK19 (ng/mL)
5
4
3
2
1
0
Pre-operative
Post-chemotherapy

FIGURE S5

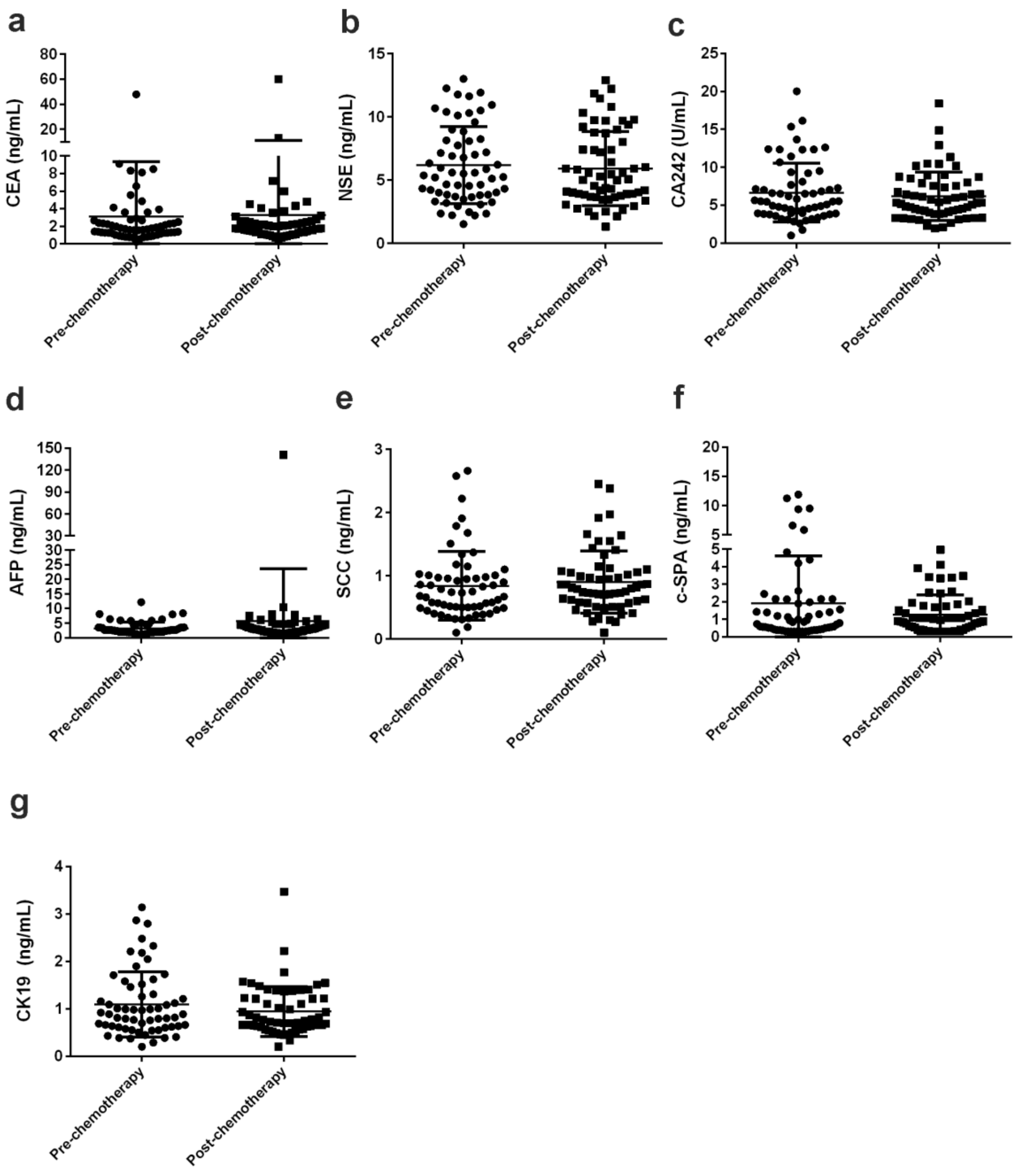
a
CEA (ng/mL)
Pre-chemotherapy
Post-chemotherapy
b
NSE (ng/mL)
Pre-chemotherapy
Post-chemotherapy
c
CA242 (U/mL)
Pre-chemotherapy
Post-chemotherapy
d
AFP (ng/mL)
Pre-chemotherapy
Post-chemotherapy
e
SCC (ng/mL)
Pre-chemotherapy
Post-chemotherapy
f
c-SPA (ng/mL)
Pre-chemotherapy
Post-chemotherapy
g
CK19 (ng/mL)
Pre-chemotherapy
Post-chemotherapy

FIGURE S6

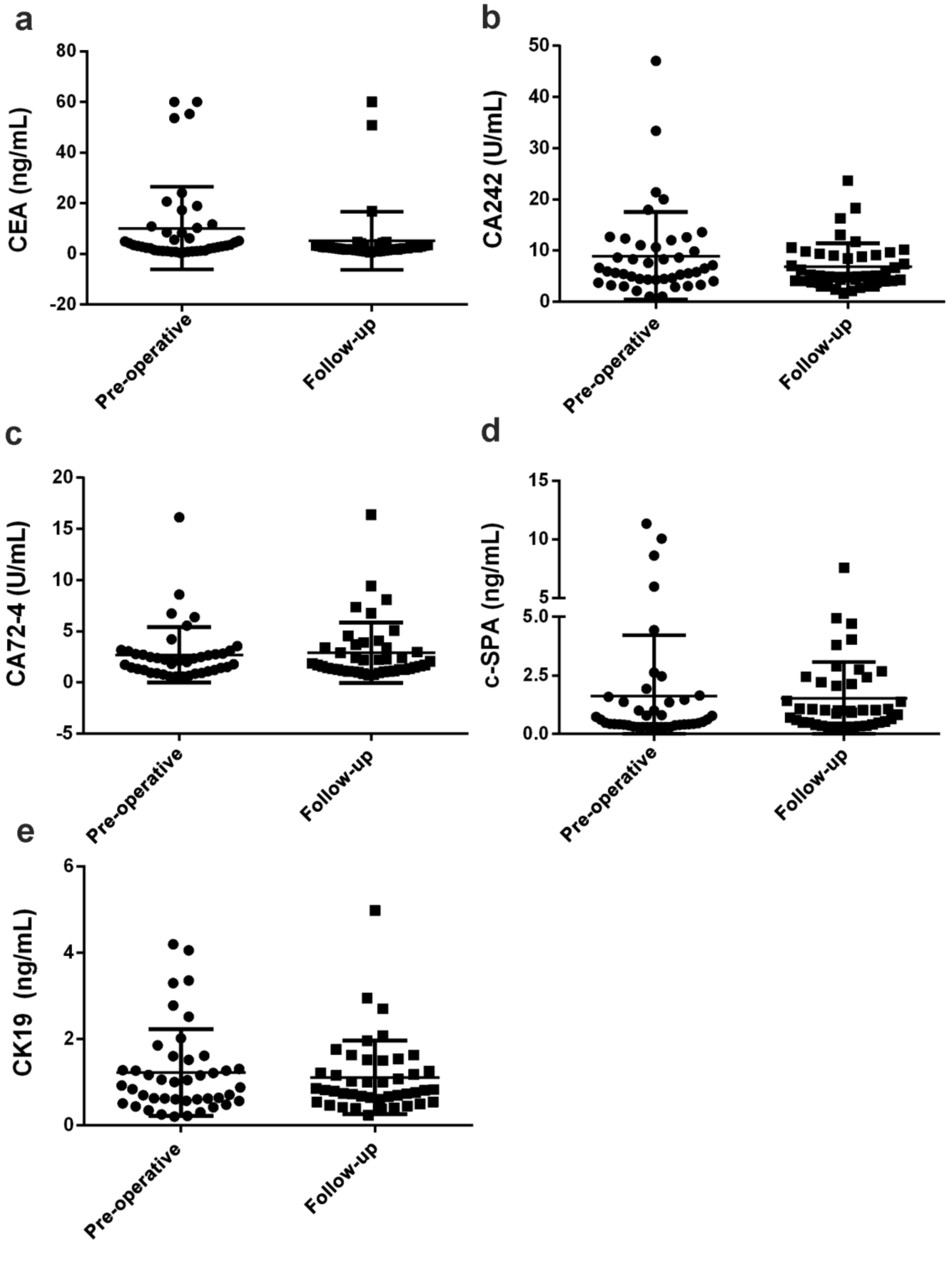
a
CEA (ng/mL)
80
60
40
20
0
-20
Pre-operative
Follow-up
b
CA242 (U/mL)
50
40
30
20
10
0
Pre-operative
Follow-up
c
CA72-4 (U/mL)
20
15
10
5
0
-5
Pre-operative
Follow-up
d
c-SPA (ng/mL)
15
10
5
5.0
2.5
0.0
Pre-operative
Follow-up
e
CK19 (ng/mL)
6
4
2
0
Pre-operative
Follow-up
